# Genetic identification of avian samples recovered from solar energy installations

**DOI:** 10.1371/journal.pone.0289949

**Published:** 2023-09-06

**Authors:** Cristian Gruppi, Peter Sanzenbacher, Karina Balekjian, Rachel Hagar, Sierra Hagen, Christine Rayne, Teia M. Schweizer, Christen M. Bossu, Daniel Cooper, Thomas Dietsch, Thomas B. Smith, Kristen Ruegg, Ryan J. Harrigan

**Affiliations:** 1 Center for Tropical Research, Institute of Environment and Sustainability, University of California, Los Angeles, Los Angeles, California, United States of America; 2 U.S. Fish and Wildlife Service, Palm Springs, California, United States of America; 3 Department of Biology, Colorado State University, Fort Collins, Colorado, United States of America; 4 Resource Conservation District, Santa Monica Mountains, Topanga, California, United States of America; 5 U.S. Fish and Wildlife Service, Carlsbad, California, United States of America; 6 Department of Ecology and Evolutionary Biology, University of California, Los Angeles, Los Angeles, California, United States of America; University of Helsinki: Helsingin Yliopisto, FINLAND

## Abstract

Renewable energy production and development will drastically affect how we meet global energy demands, while simultaneously reducing the impact of climate change. Although the possible effects of renewable energy production (mainly from solar- and wind-energy facilities) on wildlife have been explored, knowledge gaps still exist, and collecting data from wildlife remains (when negative interactions occur) at energy installations can act as a first step regarding the study of species and communities interacting with facilities. In the case of avian species, samples can be collected relatively easily (as compared to other sampling methods), but may only be able to be identified when morphological characteristics are diagnostic for a species. Therefore, many samples that appear as partial remains, or “feather spots”—known to be of avian origin but not readily assignable to species via morphology—may remain unidentified, reducing the efficiency of sample collection and the accuracy of patterns observed. To obtain data from these samples and ensure their identification and inclusion in subsequent analyses, we applied, for the first time, a DNA barcoding approach that uses mitochondrial genetic data to identify unknown avian samples collected at solar facilities to species. We also verified and compared identifications obtained by our genetic method to traditional morphological identifications using a blind test, and discuss discrepancies observed. Our results suggest that this genetic tool can be used to verify, correct, and supplement identifications made in the field and can produce data that allow accurate comparisons of avian interactions across facilities, locations, or technology types. We recommend implementing this genetic approach to ensure that unknown samples collected are efficiently identified and contribute to a better understanding of wildlife impacts at renewable energy projects.

## Introduction

The development of renewable energy solutions to replace traditional fossil-fuel energy production is likely to significantly reduce future climate change impacts, which have been identified as a threat to wildlife [1, 2]. Solar and wind energy production provided 10% of the world’s energy in 2021, and all clean electricity sources (hydropower, nuclear, wind, solar, and bioenergy) generated almost 38% of the world’s electricity in 2021, more than coal (36.5%) and natural gas (22.2%) [3]. These percentages will likely increase extensively in the coming decades as part of a global effort to meet objectives set by the Paris Climate Agreement (2015) [3]. According to the U.S. Energy Information Administration (USEIA) projections, alternative energy generation (including renewables such as solar, wind, hydroelectric, as well as geothermal and biomass) will supply 44% of U.S. electricity by 2050 [4]. Eighty-five gigawatts (GW) of new electricity are planned for release in 2022–2023, and 48% (41 GW) of this new energy will rely on solar sources [5]. Given this projected rapid expansion of renewable energy (primarily from solar and wind) development, it is crucial to analyze and understand the impacts of renewable energy facilities on wildlife to monitor, predict, and mitigate the effects of future utility-scale infrastructures.

Although renewable energy sites do show a lower impact on estimated annual avian mortality within the United States [6, 7] compared to other anthropogenically related causes, such as fossil-fuel plants [8], communication towers [9, 10], roadways [9, 11], buildings [9, 12], and predation [9, 13], increase in infrastructures may negatively affect wildlife through interactions in and around energy facilities. While previous research efforts have explored the possible effects of utility-scale renewable energy on environment [14] and wildlife [15, 16], there is currently a need to quantitatively understand both how interactions may affect populations that utilize renewable facilities (even if only for part of their life cycle) and how these interactions may change with inevitable increases in renewable energy development in the future.

Although all terrestrial wildlife may be affected by energy development, avian species may be well-suited for detailed investigation, given their relative conspicuousness at energy facilities. Two main types of interactions causing avian mortality at renewable energy facilities are currently recognized: impact trauma and exposure to concentrated solar energy (solar flux) [6, 14, 15, 17, 18]. Bird mortality due to impacts can be observed at all energy technologies as well as other anthropogenic infrastructures (e.g. window strikes), while mortality associated with solar flux is relegated only to heliostat (e.g. power tower) solar facilities [16, 18]. Mortality can also occur indirectly at all facilities, where birds become disoriented, injured, or impaired by initial interactions, reducing the capacity of individuals to resume flight, forage, or escape from predators. Traces of bird-facility interactions (i.e., the portion of those that are fatal) including intact or partial carcasses and sparse remains of avian origin called “feather spots”, can be observed and collected at facilities. And yet, even though partial or entire carcasses can be identified to species (or broader taxonomic group) by morphological characteristics *in situ*, many remains are difficult to characterize because of their small size, life stage (juvenile vs. adult), sex, or because remnants may be affected by other processes (including exposure to the elements, scavenging, and predation) before collection. Consequently, a large percentage of samples, particularly those collected at solar facilities, have been labeled as unidentified (at species or broader taxon level) in the field [19, 20]. A recent study on volant wildlife mortality at solar projects in California over a nearly thirty-year period (1982–2018), reported that 22% of fatalities could not be identified to species [7]. Based on data presented here, this percentage of unidentified avian samples can be as high as 32% (S1 Table), leading to potential sampling biases and significant data loss that could otherwise augment our understanding of the dynamics of avian species and populations at energy production facilities. Earlier studies on avian mortality at solar facilities focused only on data where morphological identifications were made and excluded samples that could not be confirmed by physical characteristics alone [6, 17, 18, 20] leading to a loss of valuable information.

**Fig 1:**
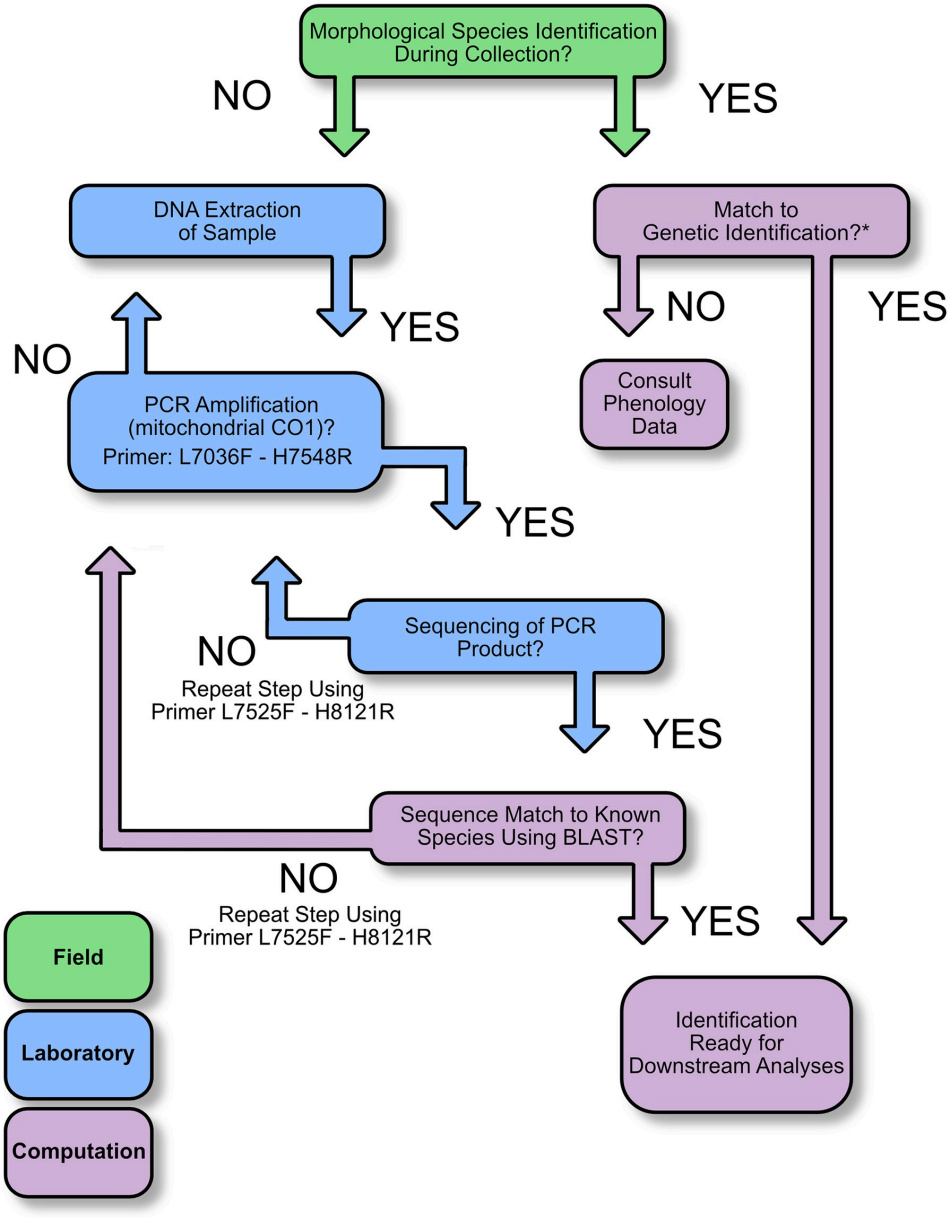
DNA barcoding analysis framework. Basic steps of the DNA barcoding analysis for the identification of samples collected at solar energy facilities. After collection, samples are processed to extract DNA, which is then amplified. PCR products are sequenced and compared to those archived on GenBank through a BLAST search, which leads to a genetic match (positive identification). Steps can be repeated using different barcoding primers when necessary. * It is strongly suggested to verify morphological identifications through the genetic approach (see “Best Practices” below) depicted here, and to include phenological data, particularly when ambiguity exists.

To address this limitation, we optimized a DNA barcoding approach using a portion of a mitochondrial gene (cytochrome c oxidase 1) to reliably identify avian samples to species. Similar approaches have previously been used to identify avian samples collected at airports [21–23], and on bats collected at wind energy facilities [24, 25]. Here, for the first time, we applied an optimized pipeline (Fig 1) to exposed, often desiccated avian samples collected in the field from desert solar facilities across a variety of solar technologies. This genetic approach can be utilized on morphologically unidentified samples, and as a check to morphology-based identifications of species in the field when the morphological assignment is ambiguous or may require verification. With this method, we aimed to determine whether unidentified samples presented a similar signal (in terms of species and community composition) as compared to those samples identified exclusively via morphology, such that an extrapolation based on morphological identifications could be used to estimate overall species and impacts on the bird community. In addition, we investigated both the reliability and limitations of each method (morphology and genetics) via a blind test to assess their accuracy in helping to identify avian remains. Using these identification methods then allowed us to compare results across three solar energy technologies to better assess the composition and biodiversity of avian communities interacting with solar installations. Lastly, we present a “best practices” guideline for including genetic barcoding analysis at solar energy facilities, or any other location where avian identifications are required, that could enhance the accuracy of community composition estimates that utilize the collected data. We envision end-users taking advantage of this reliable, efficient molecular approach for identifying or verifying any ambiguous samples of avian origin collected at energy sites, ultimately leading to more informed future management and siting strategies.

## Materials and methods

### Sample collection

Avian samples were collected at eight utility-scale solar facilities in Southern California from 2009–2021 (S1 Table). Samples found at solar installations can be categorized as either: (a) intact carcasses that are not severely decomposed and show no sign of predation, (b) partial remains, that are either entire carcasses presenting signs of predation or scavenging, or parts of a carcass limited to one location (such as a wing, leg, or other body parts), or (c) feather spots, defined as ten or more feathers (or two or more wing/tail feathers) at one location [26]. Samples were collected by authorized personnel with a SPUT (Special Purpose Utility Permit) issued to the collectors and were stored in freezers prior to shipment. Our database included samples that were either incidentally or systematically collected. Systematic mortality monitoring, coordinated with the regulatory agencies for each project, uses established survey plots randomly located within the solar arrays and along other project features such as generators, distribution/transmission (Gen-Tie) lines, and fences. Incidental finds are those found outside of these established surveys. Collections in our database occurred at facilities representing three different solar energy technologies: concentrated solar power tower (PT), concentrated solar parabolic trough (CST), and photovoltaic (PV) (S1 Table).

### DNA extraction and amplification

We used the Qiagen DNeasy Blood & Tissue Kit (QIAGEN) to isolate DNA from samples. We used at least one calamus from a wing/tail feather or up to 5 calami for body feathers for each sample. The biological material and negative controls were incubated overnight at 56°C in 180 μl Buffer ATL and 20 μl Proteinase K (600 mAU/ml solution) on a shaker to agitate the material. Dithiothreitol (10 μl, 1M DTT) was added to the extraction solution to help dissolve any keratin contained in the feathers. Samples were eluted the following day according to the manufacturer’s protocol with minor modifications. To increase DNA yield, we incubated the samples at 94°C for 5 minutes before the final elution in 100 μl of AE Buffer (10 mM Tris-HCl, 0.5 mM EDTA; pH 9.0). Given the desiccated, exposed nature of the samples collected, we quantified extractions after DNA isolation using a sensitive fluorescence-based kit (Qubit^™^ dsDNA HS Assay Kit, Invitrogen), and assessed the DNA quality (as measured by DNA fragmentation) on a subset of samples using an Agilent 2100 Bioanalyzer machine. We then assessed whether DNA quantity or quality was correlated with the ability to successfully amplify DNA and/or sequence PCR products.

Once DNA had been isolated from samples, we amplified by polymerase chain reaction (PCR) a 512 base pair (bp) target region in the mitochondrial CO1 (cytochrome c oxidase 1) gene using a pair of degenerate primers L7036 (5’-GGNACNGGNTGAACHGTNTAYCC-3’) [27] and H7548 (5’-GTDGCNGANGTRAARTADGCTCG-3’) [27]. For any samples for which the PCR amplification (or the subsequent sequencing) failed, a second 596 bp fragment adjacent to the first region was amplified using another pair of degenerate primers L7525 (5’-GTNTGRGCHCAYCAYATRTTYAC-3’) [27] and H8121 (5’-GGGCAGCCRTGRATTCAYTC-3’) [27]. We optimized the PCR settings for these two primer pairs based on previous studies [28, 29]. Reaction mixes were 15 μl in volume and contained: 5 μl of the DNA template (or H_2_O for negative controls), 0.6 μl of each primer at 10 μM concentration (for a final concentration of 0.4 μM each), 7.5 μl 2X PCR buffer (QIAGEN Multiplex PCR Buffer containing 6 mM MgCl_2_, HotStarTaq DNA Polymerase, and dNTPs), and 1.3 μl ddH_2_O. PCR conditions were 94°C for 3 min, followed by 40 cycles of: 94°C for 1 min, 54°C for 1 min, and 72°C for 1 min, followed by a final extension at 72°C for 10 min performed using a SimpliAmp^™^ Thermal Cycler (Applied Biosystems by Thermo Fisher Scientific). To optimize each step of this DNA barcode-dependent identification method, we tested and compared two sets of degenerate, universal primer pairs [27] designed to amplify distinct regions in the mitochondrial CO1 gene across several avian species. We assessed and compared the PCR performance of each degenerate primer set for its ability to amplify DNA across avian taxa and calculated a successful amplification rate (SAR) as the ratio between the number of detectable PCR products and the number of samples processed through PCR.

### Species identification

PCR products were purified by gel extraction using E-Gel^™^ SizeSelect^™^ II Agarose Gel 2% and sequenced through Sanger sequencing [30] by an external service provider (Azenta Life Sciences). Upon receiving sequencing data, we visually checked the chromatogram quality and proofread the sequences using the software Geneious Prime, Version 2022.0.1 (Biomatters Ltd). Subsequently, as reported by previous studies on species identification [31–33], we used the **B**asic **L**ocal **A**lignment **S**earch **T**ool (BLAST) on the NIH sequence database (GenBank) to align sequences with potential matches among the millions of publicly available sequences in this database (https://blast.ncbi.nlm.nih.gov/Blast.cgi). We identified samples by comparing the sequence (query sequence) from our target sample with those (subject sequences) contained in GenBank’s comprehensive reference database. We primarily used Mega BLAST (a BLAST filter tool) as an option for searching to prioritize based on highly similar sequences, and we subsequently considered higher sequence identity and higher query sequence coverage as primary parameters to indicate a higher probability of taxonomic identification [34].

We set a general match criterion of 95% as a threshold of nucleotide identity for a species assignment, including both positive and questionable identifications (Table 1). In cases of multiple high homology matches, we considered the highest sequence coverage percentage as a preferential parameter for final species identification. Since information on the date of collection and GPS coordinates were available, we additionally verified our identifications leveraging taxon-specific phenology and habitat range information (i.e., timing of species movements) from eBird (https://ebird.org/home) and Birds of the World (https://birdsoftheworld.org/bow/home). Based on the BLAST criteria we utilized to determine species identification, we then categorized the genetic identifications into three distinct groups: “positive identification” (where sequence identity was >97% for a species), “questionable identification” (for which multiple high homology matches occurred or GenBank search showed lower BLAST match metrics), and “no identification” (Table 1). Lack of identification could be due to a number of reasons, including inefficient PCR amplification, ineffective sequencing, lack of matching sequences in GenBank, or inconsistencies in species phenology (Fig 1). It should be noted that we genetically identified samples representing waterfowl that, in some cases, matched two species that are undistinguishable through DNA barcode sequences used in this study. These matches in identity for the CO1 region are due to the fact that in some cases, closely-related, often hybridizing, sister species share identical mitochondrial DNA, such as the Blue-winged Teal (*Spatula discors*) and the Cinnamon Teal (*Spatula cyanoptera*), or the Western Grebe (*Aechmophorus occidentalis*) and Clark’s Grebe (*Aechmophorus clarkii*). In these cases, both sister taxa identifications are reported in the dataset (Dryad Accession: https://doi.org/10.5061/dryad.fbg79cp1g).

**Table 1:** Categorization of samples identified via genetic approach.

Category	PCR	Sequence HQ%	BLAST Hits	BLAST Coverage	BLAST Identity	Phenology	Num. of Samples	Percentage
Positive Identification	Yes	> 20%	Yes	> 50%	> 97%	Consistent	668	84.1%
Questionable Identification	Yes	< 20%	Yes	30%–50%	95% < X < 97%	Unlikely	71	8.9%
No Identification	Yes/No	< 20% or N/A	No or N/A	< 30%	< 95%	Inconsistent	55	6.9%

Description of sequencing, BLAST (Basic Local Alignment Search Tool), and phenological parameters used to categorize samples and corresponding number of avian samples listed in each of three defined categories after processing through DNA barcode analysis. Sequence HQ% represents the percentage of bases in a sequence showing high-quality resolution in the variant calling process. Samples in this table were avian remains, primarily feather spots, of unknown species origin.

### Verification of samples identified via morphology

As a verification of this DNA barcode approach, we performed a blind test on a subset of 48 specimens that had been positively identified in the field by morphology alone (Dryad Accession: https://doi.org/10.5061/dryad.fbg79cp1g). This subset was selected by colleagues in an effort to cover a broad range of taxa, seasons, locations, and solar energy technologies. We genetically analyzed these specimens (labeled Specimens 1–48) without knowing these identifications, and then compared our genetics-based identifications with those obtained in the field based solely on morphology.

### Identification to individual

Finally, we were able to estimate how many individuals were part of the full dataset collected using unique sequence data. As individual sequencing data is produced as part of the barcoding process for each sample, we aligned and compared sequences from across our entire dataset, particularly from samples that were recovered either during the same week (or on the same day), and at the same location. For those samples that shared identical DNA sequences, we considered these samples as potentially originating from the same individual, such that they could artificially inflate the reported number of birds impacted at solar facilities.

### Comparison of identification methods

To determine the types of avian samples that may be less likely to be identified in the field, we compared and statistically assessed the field-based identifications to those obtained through the genetic method. We exclusively used raw data with no adjustment factor for search methods, differential collection effort and efficiency, ability to identify samples, and carcass persistence tests, either within or across facilities, and excluded samples that could not be identified by either method. We then categorized the samples into three broad groups based on common taxonomic and ecological characteristics: Terrestrial Birds (non-passerines such as raptors, landfowl, and hummingbirds), Songbirds (passerines), and Waterbirds (ducks, geese, gulls) (see S2 Table for full list of included Orders), and compared patterns of observed avian identifications recovered by each method using a χ^2^ test for proportions. We also compared bird community compositions at Order and Family level. Finally, after integration of morphological and genetic identification data, we compared the proportions of the three avian groups listed above across solar energy technologies and tested for significant differences using a χ^2^ test for proportions.

## Results

### Sample collection

Of the 4,383 samples collected, 794 (accounting for 18.12%) were listed as unknowns, defined as those samples for which any scale of ambiguity existed when morphological identifications were made (i.e., listed as “unknown bird”, “unknown duck”, or “unknown hummingbird”). These samples were the subject of subsequent processing using the laboratory methods presented below, and hereafter referred to as “genetic” identifications, as compared to the 3,589 samples that were “morphological” identifications in the field.

### DNA extraction and amplification

We processed 794 unidentified samples collected from eight utility-scale solar sites collectively representing three different solar energy technologies (two CST, one PT, and five PV sites) (S1 Table). Despite the variability observed amongst samples, the mean of extracted DNA across all samples was 1.56 ± 2.67 μg per sample (respectively, 2.39 ± 3.73 μg from PV, 2.62 ± 2.92 μg from CST, and 0.43 ± 1.28 μg from PT sites), and we extracted at least 10 ng of total DNA from 90.1% of the samples. We did not find any correlation between DNA quantity (R^2^ = 0.008) and/or quality (visual inspection of the fragmentation profile) and success rate of PCR amplification or sequencing, suggesting that even exposed and/or degraded DNA can produce genetic data for subsequent analyses. We obtained SAR values of 85.6% and 79.4%, respectively, for the L7036-H7548 and L7525-H8121 primer sets, and then discretionally assigned the primer pair with the higher SAR as primary (CO1 primer set 1) and the remaining primers as secondary (CO1 primer set 2) (Fig 1).

### Species identification

On average, we were able to sequence 481bp for the L7036-H7548 amplicon (CO1 primer set 1), and 550bp for the L7525-H8121 amplicon (CO1 primer set 2). We sequenced the amplicons using both corresponding forward and reverse primer and they showed no preferential ability to reliably sequence the target amplicon. More importantly, we observed no discrepancy in downstream identifications. This suggests that use of either forward or reverse as a sequencing primer is a cost-effective and time-saving step for the species assignment process. Using both forward and reverse primers, however, may provide an increased sequence length that may help to identify mitochondrial DNA sequences at the individual level in some cases (e.g., when discriminating between closely-related individuals).

Moreover, we investigated the ability of amplicons’ sequences, from mitochondrial DNA regions amplified by both primer sets, to probabilistically match to sequences deposited in the GenBank database as well as to assess the confidence of species identifications. For each sample of different species successfully amplified with both primer sets, we compared each sequencing primer using the highest BLAST percentage identity between the query sequence and the subject sequence as a proxy metric for probabilistic assignment to a species. We then calculated the similarity coefficient per primer set for all the samples analyzed. Using CO1 primer set 1, we obtained a mean identity of 99.03%, while CO1 primer set 2-derived sequences had a mean identity match of 97.78%. Finally, we compared the species identifications obtained through L7036-H7548 and L7525-H8121 primer pairs only on high-quality solar samples defined as: (i) sequencing quality (HQ%) higher than 20%, (ii) BLAST percentage coverage higher than 50%, and (iii) BLAST percentage identity higher than 97% for both primer pairs. No species discrepancies were observed, suggesting that sequencing a single PCR product is sufficient on most samples to genetically identify them at a species level.

When we further tested DNA from samples that failed to be reliably identified to species using CO1 primer set 1, due to either lack of PCR product or lack of a consistent match in the GenBank database, we found that performing an amplification step with CO1 primer set 2 yielded a higher identification success rate than simply repeating the PCR using the first primers. We were able to identify species for over 50% (53 of 103) of samples that failed the first round of identification due to lack of reliable hits in GenBank, and an additional 14 samples out of 37 (37.8%) that failed the first PCR amplification when the primer set was switched. In contrast, we only identified an additional 7.7% of individuals when we repeated the PCR amplification using the same primer set for both rounds.

We were able to identify to species a total of 668 out of 794 (84.1%) previously unidentified samples with a confidence probability higher than 97%, and an additional 71 samples (8.9% of total) with an identification probability higher than 95% (Table 1, Dryad Accession: https://doi.org/10.5061/dryad.fbg79cp1g). Only 6.9% (55 samples) of the morphologically unidentified samples could also not be genetically identified, and require further investigation to determine the species to which they belong. Through this optimized DNA barcoding analysis, we expanded the number of samples that can be effectively included in wildlife monitoring by a total of 17% across all locations and years (18% unidentified *prior to*, as compared to 1% unidentified *after*, the genetic approach was implemented).

### Verification of samples identified via morphology

We performed a DNA barcode analysis on a subset of 48 samples (Dryad Accession: https://doi.org/10.5061/dryad.fbg79cp1g) that had previously been morphologically identified to species in the field, but whose identifications were withheld from us, as a blind test verification (S3 Table). Of these, 42 matched in identification between morphology and genetics, with six (12.5%) showing taxonomic discrepancies between morphological and genetic identifications (Table 2).

**Table 2:** Discrepancies between morphological and genetic identifications.

	Morphological Identification	Genetic Identification
Specimen 12	Great-tailed Grackle (Quiscalus mexicanus)	Brewer’s Blackbird (Euphagus cyanocephalus)
Specimen 16	House Finch (Haemorhous mexicanus)	Brown-headed Cowbird (Molothrus ater)
Specimen 18	Mountain Bluebird (Sialia currucoides)	Western Bluebird (Sialia mexicana) / Mountain Bluebird (Sialia currucoides)
Specimen 20	Bell’s sparrow (Artemisiospiza belli)	Black-throated Sparrow (Amphispiza bilineata)
Specimen 27	Blue-gray Gnatcatcher (Polioptila caerulea)	Black-tailed Gnatcatcher (Polioptila melanura)
Specimen 35	Hooded Merganser (Lophodytes cucullatus)	Red-breasted Merganser (Mergus serrator)

These six discrepancies represent 12.5% of the total screened. Blue indicates a confirmed identification, red indicates misidentification, and yellow represents ambiguity between two closely-related species (questionable identification, see text for details).

Of these six mismatches, five appeared to be errors in morphological identifications performed in the field, given that identical (and phenologically congruent) genetic matches were found for these samples in the GenBank database. For these mismatches, we were further able to confirm morphological identifications were incorrect by utilizing remnants, photographs, and field notes from the laboratory where the samples were stored. These investigations confirmed that the original field identifications were likely erroneous. The remaining mismatch was the result of an ambiguous genetic identification of two closely-related species, the Mountain Bluebird (*Sialia currucoides*) and Western Bluebird (*Sialia mexicana*) (Table 2). Of note here is that this latter mismatch is objectively identified and reported, relegated to sub- or sister species (unlike some morphological mis-identifications), and likely to be resolved as further data is collected and uploaded to GenBank. In scenarios where mtDNA is identical between sister taxa, such as in closely-related hybridizing species (e.g. ducks), morphology can be complimentarily utilized for identification.

### Identification to individual

An additional advantage of using this optimized genetic approach is the ability to go beyond species identification and, in many cases, assign collected samples to an individual. We aligned and compared sequences from different samples with matching species identifications sampled during the same week (or day), at the same solar facility, to determine whether they could have potentially originated from the same individual. We estimate that a maximum of 6% (275 of 4,383) of the feather spot/remnants collected in this study could theoretically represent the same individual. This estimate is further reduced to 3% (137 of 4,383) when only the sequences from samples collected on the same day are considered as a more stringent criterion for match. In this study, the vast majority of samples collected represent unique individuals, rather than the biological remains of a single individual being found at a variety of locations.

### Comparison of identification patterns

**Fig 2:**
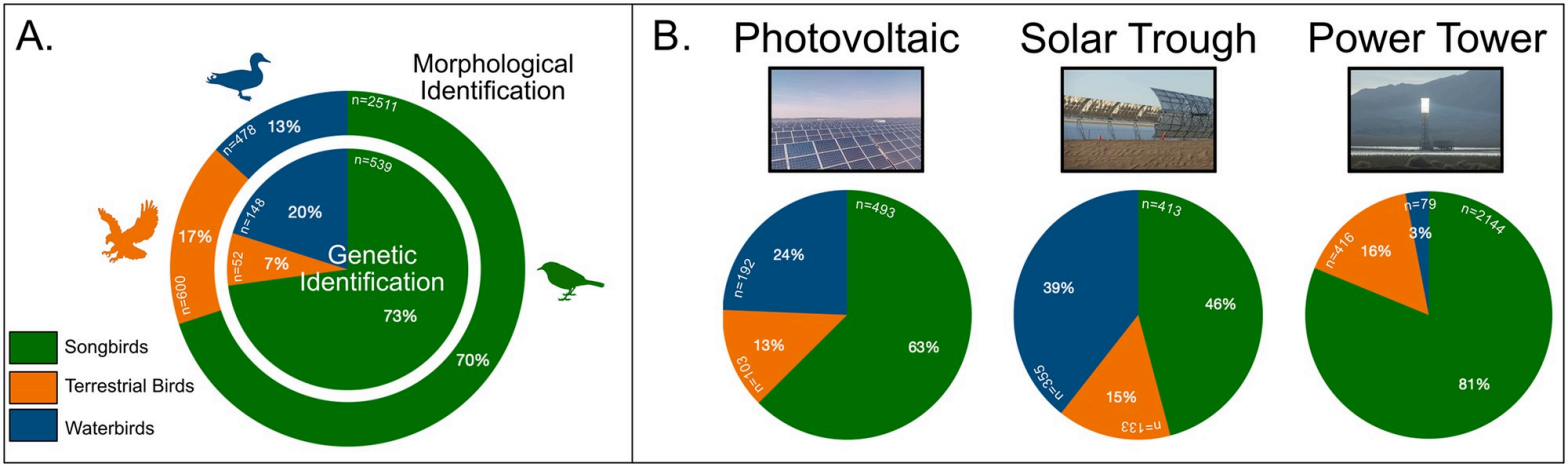
Proportions of avian communities. Comparison of the overall proportions of avian community categorized as Terrestrial Birds, Songbirds, and Waterbirds. (A) Proportions are significantly different (χ^2^ = 57.96, p<0.00001, n = 4,328) between genetic identifications (inner circle) and morphological identifications (outer circle). (B) Differences in avian group proportions across technology type (example photo above each chart). A significant difference (χ^2^ = 811.84, p<0.00001, n = 4,328) in bird group proportions was observed by technology type.

We tested whether remains left from particular avian groups might make it less likely that they be identified in the field, due either to similar morphological traits or to the poor quality of remains. After categorizing samples into three broad groups (Terrestrial Birds, Songbirds, and Waterbirds) based on their common characteristics (S2 Table), we compared the genetic taxonomic identifications of unknowns with those carried out by morphological assessment. We found that genetic and morphological identifications showed a significantly different taxonomic distribution of avian species interacting with solar infrastructures (χ^2^ = 57.96, p<0.00001, n = 4,328; Fig 2A and S1 and S2 Figs). For instance, Terrestrial Birds represented 17% of the total morphologically identified samples, yet only 7% of unknowns identified via genetic analysis. In contrast, while Waterbirds comprised only 13% of the total identified samples via morphology, they represented 20% of previously unidentified samples that were genetically identified. Songbirds represented the largest proportion of identifications using both methods, accounting for 73% and 70% for DNA barcode and morphological identifications, respectively.

Combining data from both methods, we also observed significant differences in the proportions of the three bird groups at facilities using different solar energy technologies (χ^2^ = 811.84, p<0.00001, n = 4,328; Fig 2B). A larger percentage of Waterbirds were found at photovoltaic and solar parabolic trough sites (24% and 39%, respectively), in contrast to the one solar power tower site where the vast majority of samples (81%) were Songbirds. The percentage of Terrestrial Bird samples was comparable across all solar energy technologies and locations. We found a similar avian community distribution, but different proportions, across solar technologies when analyzing data separately for the morphological and genetic identifications (S3 Fig).

Finally, we were also able to confirm the identification of 12 species based on genetics that had not previously been identified in our dataset using morphological methods alone (S4 Fig). These included the American Herring Gull (*Larus argentatus smithsonianus*), California Quail (*Callipepla californica*), Phainopepla (*Phainopepla nitens*), Snowy Plover (*Charadrius nivosus*), Blue Grosbeak (*Passerina caerulea*), Fox Sparrow (*Passerella iliaca*), American Redstart (*Setophaga ruticilla*), Grasshopper Sparrow (*Ammodramus savannarum*), Hammond’s Flycatcher (*Empidonax hammondii*), Red Crossbill (*Loxia curvirostra*), Palm Warbler (*Setophaga palmarum*), and Willow Flycatcher (*Empidonax traillii*), and were based on similarity matches to published sequences available on GenBank greater than 98%.

## Discussion

Understanding the spatial and temporal patterns of interactions of wildlife with renewable energy infrastructure, and how these may change under rapid climate shifts and anthropogenic land transformation, is essential for effective conservation in the future. To better understand the impacts to wildlife of utility-scale solar energy sites, federal and state agencies proposed an Avian-Solar Science Coordination Plan [35] to prioritize information needed to investigate bird-solar facility interactions. Specifically, the Avian Solar Working Group (ASWG, http://www.aviansolar.org), a collaborative group of NGOs, academics, and solar industry representatives whose aim is to investigate how birds interact with solar facilities, identified an interest in understanding the composition of feather spots and other unidentified avian samples to distinguish the species and number of individuals from which they originate [36]. Previous studies have relied solely on data where morphological characteristics were used to identify such remains of avian origin [6, 17, 18, 20]. Yet, these studies excluded samples that could not be distinguished by morphology due to small size, life stage, sex, similarity of closely-related species, poor quality of the sample because of predator or scavenger activity, and/or exposure to the elements prior to collection. These bird samples (primarily feather spots), previously left unidentified, represent a significant addition to the scientific record that can help address some of the key questions concerning solar energy facilities and their effects on wildlife.

Myriad questions remain regarding how wildlife may interact with renewable energy facilities, and to address any of them, the collection of data is vital. Data collection is an intensive endeavor, both for regulatory agencies and for solar energy companies, and its related soft costs are often passed on to the consumers, thereby reducing the perceived benefits of a shift to renewable energy production. It becomes clear, then, that collection of samples at solar energy sites needs to be performed as efficiently and standardized as possible to ensure the maximum amount of accurate data can be included in analyses using the minimum amount of time and effort and to make analyses comparable across sites and over years [26, 37–40].

We optimized an efficient DNA barcoding-based approach to identify to species samples collected at solar energy facilities, allowing their inclusion in the scientific record for subsequent analyses and monitoring studies. This genetic method (Fig 1) consists of straightforward, easy-to-use laboratory methods that can be performed rapidly (~48 hours), on standard equipment with minimal training. These features make this genetic analysis attractive for a broad range of applications. In this study, we opted for Sanger sequencing instead of using a Next Generation Sequencing (NGS) pipeline because of its advantageously low (0.001%) sequencing error rate [41, 42] compared with NGS platforms [43]. In addition, the amplicons’ length (>500 bp) to be sequenced is more suitable for Sanger sequencing than NGS [44], and reliable universal degenerate CO1 primers designed specifically for birds [27] are available. Finally, it is easy to visually check and easily proofread the sequences’ chromatograms using intuitive GUI software (e.g., Geneious (Biomatters Ltd)). Given that an NGS approach could be an extremely useful and a powerful tool to efficiently obtain large amounts of genetic data in a cost-saving and time-saving way, however, suggests it should be given further consideration, particularly when thousands of samples need to be analyzed and/or if high throughput is paramount.

Although our study was restricted to a small geographic region (the Southern California Desert) and a single type of renewable energy (solar), this DNA barcoding approach is broadly applicable to any geographic region, and we anticipate the ability to identify feather spots/remains across various types of alternative energy facilities (e.g., wind, solar, geothermal), and technologies, or to quantitatively compare these results with those recovered under other circumstances (e.g., cat kills, window strikes, airport collisions, or natural disasters).

We were able to accurately identify a total of 739 (of 794, 93%) previously unidentified samples to species, and in most cases, to individual. Interesting to note is the fact that we did not observe any correlation between successful species assignments obtained and sample DNA quantity or quality, demonstrating how powerful this genetic approach can be for identifying biological samples exposed to harsh conditions. The ability to reliably process DNA extracted from samples collected in the field led to a higher number of identifications of collected samples, which significantly altered the proportion of birds estimated to be impacted at facilities (Fig 2A and S1 and S2 Figs). By using this genetic approach, we increased the species-level data extracted from monitoring by ~17%, which can lead to better datasets used in downstream analyses.

When blind-testing 48 samples collected and identified at PV and CST facilities during incidental monitoring to verify DNA-based identifications, we found instances (12.5% of the total tested) where morphological and genetic identifications were incongruent (Table 2). For most mismatches (5 out of 6), we found that the genetic identification, based on an identical match to a previously identified sequence available in GenBank, was more likely correct than the morphological one. Misidentification of feather spots/remains at the species level based on morphology alone may occur for several reasons, including: a) the experience and knowledge of the identifier, b) *a priori* knowledge biases concerning the species (for instance, when a known range falls outside of the collection location), c) difficulty in discriminating juveniles of some species from adults of another, or d) problematic identification of closely-related species (particularly females), especially when the samples are compromised. This result highlights the additional value of the DNA barcoding method presented here as an accurate, easy-to-apply verification tool to reduce ambiguity or uncertainty in identifications.

Analysis of identifications revealed that the genetically identified samples did not simply reflect the larger species composition identified in the field via morphology, but instead provided additional insights on the proportions of birds affected at a facility (Fig 2A). The genetic identifications obtained also span a broad range of taxa (S1 and S2 Figs), suggesting that difficulties in morphological identifications are not relegated to specific taxa that might be particularly hard to identify via morphological features, or that may be more frequently or thoroughly scavenged. These results demonstrate that identification via morphology should not be considered an exhaustive tool for the investigation and estimation of wildlife interactions with solar energy facilities, and may in fact introduce biases. For instance, comparing the proportions of Terrestrial Birds, Songbirds, and Waterbirds obtained by either genetic or by morphological method, showed two distinct distribution patterns—Waterbirds were consistently under-represented and Terrestrial Birds over-represented in the group of samples that were morphologically identified. This overall pattern was consistent across types of solar technology at the collection sites, and it highlights the utility of DNA barcode in adding new information to, and enhancing the accuracy of, a collected dataset (S3 Fig).

The DNA barcoding method, despite its advances, still has limitations. This method is dependent mainly on DNA from exposed samples, and successful amplification may depend on habitat, climate, collection procedures, and exposure conditions of the samples collected [45]. Of the 4,383 total samples collected as part of this work, 55 samples could not be identified using this genetic identification process. However, this represents a much smaller percentage of unknowns (1.2%) of the total samples collected compared to the mean percentage of unidentified samples when morphology alone is used (18.1%), again suggesting the importance of including DNA-based analyses. This high recovery and identification rate is due in part to: 1) CO1 degenerate primers designed to efficiently amplify their target mitochondrial region across bird taxa [27], 2) the targeting of well-studied DNA regions in the avian CO1 mitochondrial gene, widely used for DNA barcoding studies [46–50], other applications [51] or biological inferences [52], and 3) representation of a large majority of North American avian species that could be found at facilities already represented in the GenBank sequence database. Similarly high rates may not be feasible when 1) non-avian species are targeted, for which less genetic information to compare unidentified samples to may be available, or 2) geographic regions are sampled where fewer avian species have been sampled or sequenced. These potential limitations, however, are likely to be further reduced as additional sequences and genomes are added to online databases in the future.

An inherent limitation in such DNA barcoding methods is the possibility of obtaining multiple high similarity matches using an online database (i.e., GenBank) for different species. Even when conservative in performing analyses, multiple matches to different species can still occur. To rule out some of these matches, we were also able to use information about the phenology of a species, including the presence in a specific habitat range and ecological data of an assigned species, to assist in identifications. Nevertheless, a notable advantage of this genetic method is that even when a sample cannot be unambiguously identified, the uncertainty can be quantified based on the probability of identity match to sequences found on GenBank. In contrast, for identifications based on morphology, there is no quantitative way to determine how confident one might expect to be for any particular sample, and this confidence may vary according to the quality of the sample collected, observer bias, experience, or knowledge of the species in a given area.

An additional potential advantage of leveraging genetic data is the possibility to distinguish individuals beyond the species level, which could help better estimate the total number of individual birds interacting with energy facilities. It has been hypothesized, for instance, that several feather spots may originate from a single individual at a solar energy facility, such that the total number of individuals affected by activities may be overestimated in some cases. We found in our study, that a maximum of 6% of all feather spots collected could theoretically have originated from the same individual. However, the true percentage is likely to be even smaller because different individuals may share identical mitochondrial DNA, especially flocking birds found together. These results suggest that until otherwise supported, individual feather spots collected at solar facilities are best assumed to represent a single individual. It should be noted here, however, that all samples studied were collected only at solar facilities (that employed a variety of technologies), and the same assumption should not be made for different renewable energy technologies (e.g., wind) that are likely to vary on the number of feather spots left from individuals based on the dynamics of bird/infrastructure interaction.

All analyses presented here are based on raw data, and no mathematical adjustments were made to correct for potential collection or persistence biases within or across facilities which, if made, may help to more accurately estimate avian fatality rates at solar energy sites [18, 53, 54]. One such feature that could contribute to detection biases is the size of a bird, such that raw data may globally underestimate bird groups with a particular body size, but this misrepresentation is likely to be consistent across all the sites, technologies, and years, such that comparisons of proportions should remain similar. These and other corrections were excluded given the difficulty with consistently estimating them across facilities, which can then lead to incorrect estimators that may greatly affect conclusions on impacts to populations or species [7, 55]. Also of note is that the U.S. Desert Southwest, where our samples were collected, is a largely contiguous ecosystem with consistent environmental characteristics across seasons and years amongst sites, and caution should be used when extrapolating to other regions or ecosystems. Lastly, we assume here that all samples recovered from facilities represent individuals that interacted with infrastructure in a way that led to mortality, but a greater understanding of cause of mortality and/or whether predators or scavengers might utilize facility grounds to process prey items is warranted.

Several species identified through DNA barcodes that were not identified morphologically require further investigation due to their threatened conservation status. In particular, we genetically identified the Snowy Plover (*Charadrius nivosus*, three individuals) and the Willow Flycatcher (*Empidonax traillii*, one individual). The Snowy Plover is a waterbird listed as a Near Threatened (NT) species since 2014 by The IUCN Red List of Threatened Species, and its global population is decreasing [56]. The Willow Flycatcher is a Neotropical migrant bird for which four subspecies have been ecologically [57, 58] and genetically [59–62] identified. One of these, the Southwestern Willow Flycatcher (*Empidonax traillii extimus*) is a U.S. federally endangered subspecies [63] and needs particular attention due to its small population size. Combining our genetic results with “genoscape” data, derived from a method developed to distinguish and map further subpopulations of a given species based on genomic variation across a geographic habitat [64], to assess the genetic vulnerability of an avian population facing the occurring climate change [55, 65, 66], and to investigate the migratory behaviors during the entire life cycle [67], would allow a greater understanding of the population-specific impacts that energy facilities may be having on these subdivisions of a species. These examples support the benefit of collecting DNA whenever possible to most efficiently characterize avian species and population impacts at facilities.

Based on our work, we suggest a set of “Best Practices” for the collection and identification of samples of avian origin at solar energy sites, or anywhere where the collection of material from avian mortality events may be of interest:

1. Collect samples in a timely, coordinated manner, and record any and all information about the collected sample (e.g. GPS coordinates, time of collection).
2. Include a preliminary identification of each sample based on morphological characteristics (when they are present).
3. For any unidentified or ambiguous samples found in (2), submit samples for identification using the genetic approach outlined above.
4. Use morphological and genetic data collected in (2) and (3) to verify identifications before including in downstream analyses (Fig 1).

Our results demonstrate that utilizing a molecular approach to identify unknown samples, as well as to verify those samples morphologically identified, helps to alleviate misidentifications, sampling errors, and the loss of information. These reductions in errors and losses can optimize sample collection efforts and maximize the amount and quality of total data collected on birds impacted by solar energy production sites. The contribution of information from these previously unknown samples will lead to a higher resolution and more accurate assessment of how species and populations interact with or are impacted by solar energy facility infrastructures. Such assessments are essential to ensure that science-based decisions can be made by the solar energy industry, state and federal agencies, and conservation groups, and can be utilized to inform and manage existing and future solar energy installations while implementing the best mitigation practices possible.

## Supporting information

S1 Fig Comparison of the proportions of samples categorized by order between morphological and genetic identifications. Comparison of the overall proportions of avian community categorized by taxonomic Order. Proportions are different between genetic identification (inner circle) and morphological identification (outer circle). Colors are similar to the three major categories (Songbirds, Terrestrial Birds, Waterbirds) presented in Fig 2 (main text), and total number of individuals belonging to each Order identified using morphological and genetic method are presented to the right of the figure.
(TIF)

S2 Fig Comparison of the proportions of samples categorized by family between morphological and genetic identifications. Comparison of the overall proportions of avian community categorized by taxonomic Family. Proportions are different between genetic identification (inner circle) and morphological identification (outer circle). Colors are similar to the three major categories (Songbirds, Terrestrial Birds, Waterbirds) presented in Fig 2 (main text), and total number of individuals belonging to each Family identified using morphological and genetic method are presented to the right of the figure.
(TIF)

S3 Fig Comparison of the two methods of species identification: Genetics versus morphology by solar energy technology. Proportions of bird groups (Terrestrial Birds, Songbirds, and Waterbirds) of all samples in the dataset presented by the solar technology installed at the collection sites. Photovoltaic (PV), Concentrated Solar Through (CST), Concentrated Solar Power Tower (PT).
(TIF)

S4 Fig Avian species (n = 12) in our dataset that were only identified via DNA barcode analysis. All photographs used are under the Creative Commons (CC) License.
(TIF)

S1 Table List of solar sites and collected samples. Numbers and sites where samples were collected. Three types of technology are represented at these sites: Photovoltaic (PV, n = 5), Concentrated Solar Power Tower (PT, n = 1), and Concentrated Solar Power Parabolic Trough (CST, n = 2).
(PDF)

S2 Table Avian orders included in each of the described three major categories. Samples were categorized into Terrestrial Birds, Waterbirds, and Songbirds for this study.
(PDF)

S3 Table Full list of blind-tested avian specimens. Avian specimens used in the blind test. Specimens that did not match between morphological and genetic methods are highlighted by an * (see Table 2).
(PDF)
