# Supplementary figures and images for: Genetic identification of avian samples recovered from solar energy installations
Source: PLoS One. 2023 Sep 6;18(9):e0289949. doi: 10.1371/journal.pone.0289949 (PMC10482291; doi:10.1371/journal.pone.0289949)

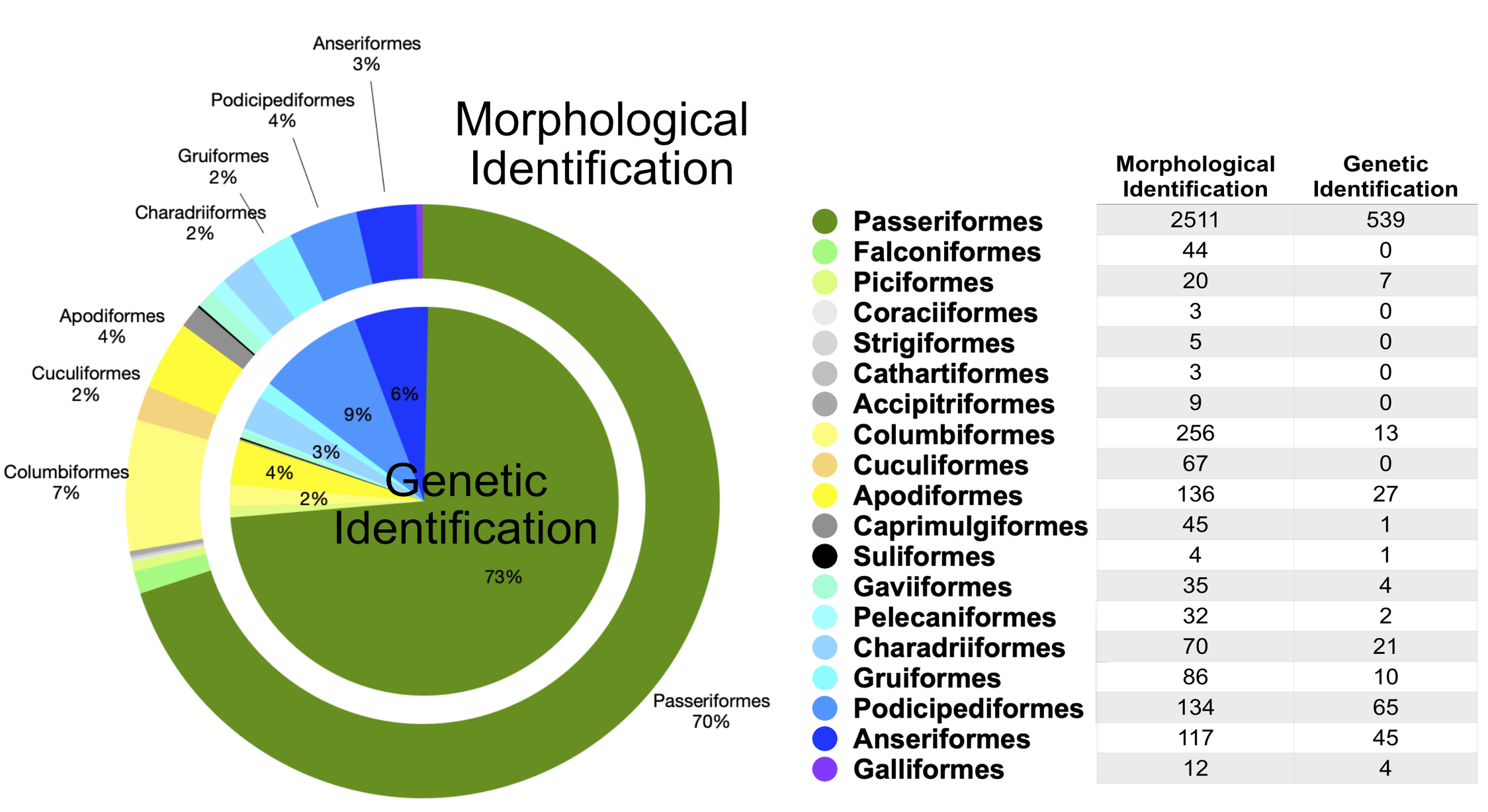

Supplement: S1 Fig — Comparison of the overall proportions of avian community categorized by taxonomic Order. Proportions are different between genetic identification (inner circle) and morphological identification (outer circle). Colors are similar to the three major categories (Songbirds, Terrestrial Birds, Waterbirds) presented in Fig 2 (main text), and total number of individuals belonging to each Order identified using morphological and genetic method are presented to the right of the figure. (TIF) [file pone.0289949.s001.tif]

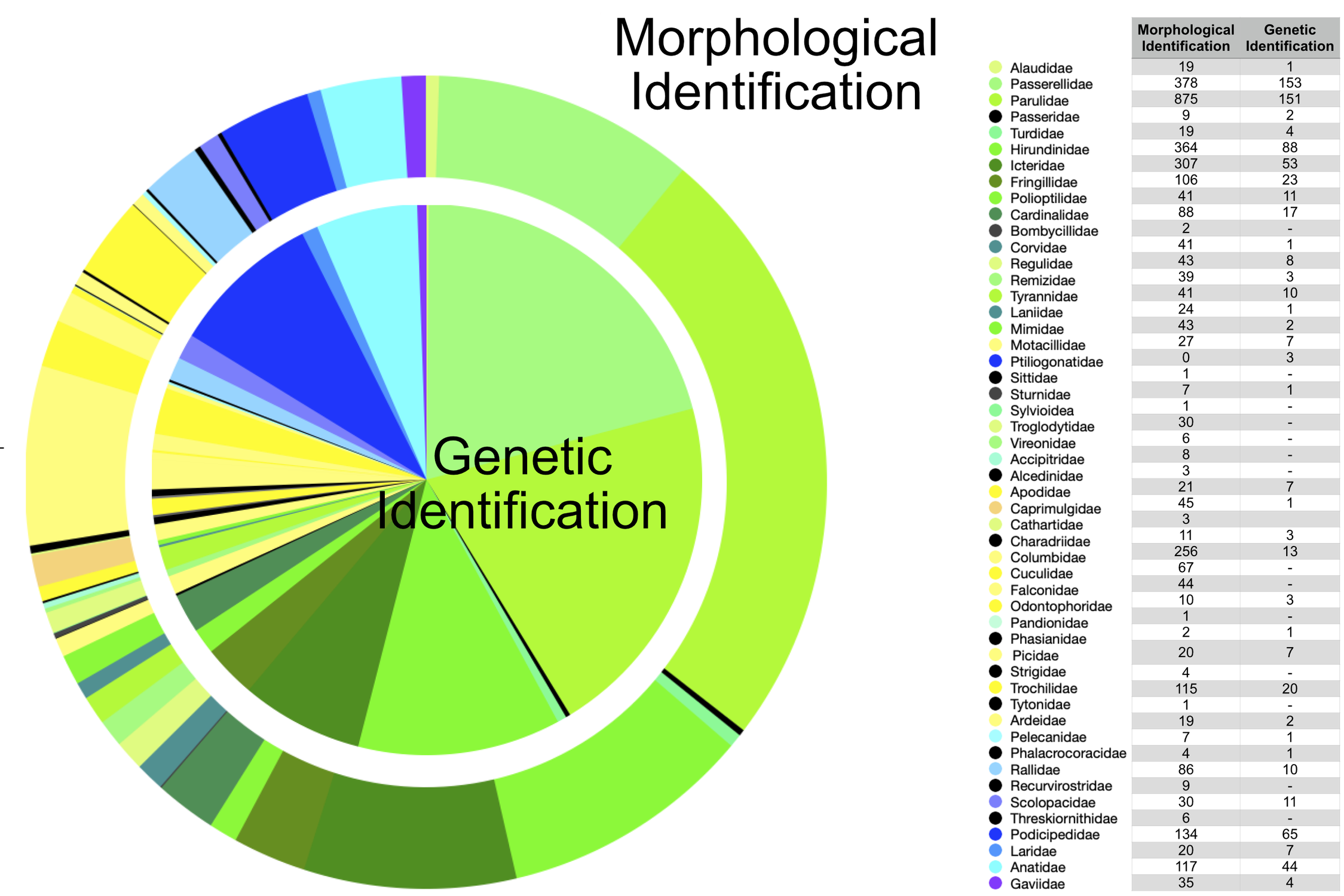

Supplement: S2 Fig — Comparison of the overall proportions of avian community categorized by taxonomic Family. Proportions are different between genetic identification (inner circle) and morphological identification (outer circle). Colors are similar to the three major categories (Songbirds, Terrestrial Birds, Waterbirds) presented in Fig 2 (main text), and total number of individuals belonging to each Family identified using morphological and genetic method are presented to the right of the figure. (TIF) [file pone.0289949.s002.tif]

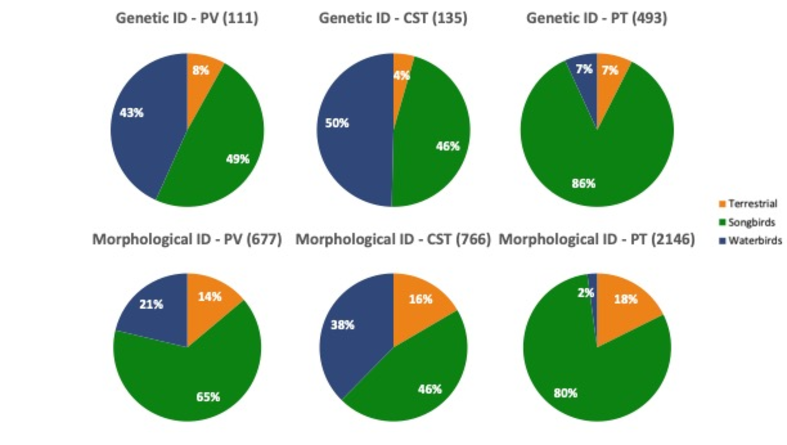

Supplement: S3 Fig — Proportions of bird groups (Terrestrial Birds, Songbirds, and Waterbirds) of all samples in the dataset presented by the solar technology installed at the collection sites. Photovoltaic (PV), Concentrated Solar Through (CST), Concentrated Solar Power Tower (PT). (TIF) [file pone.0289949.s003.tif]

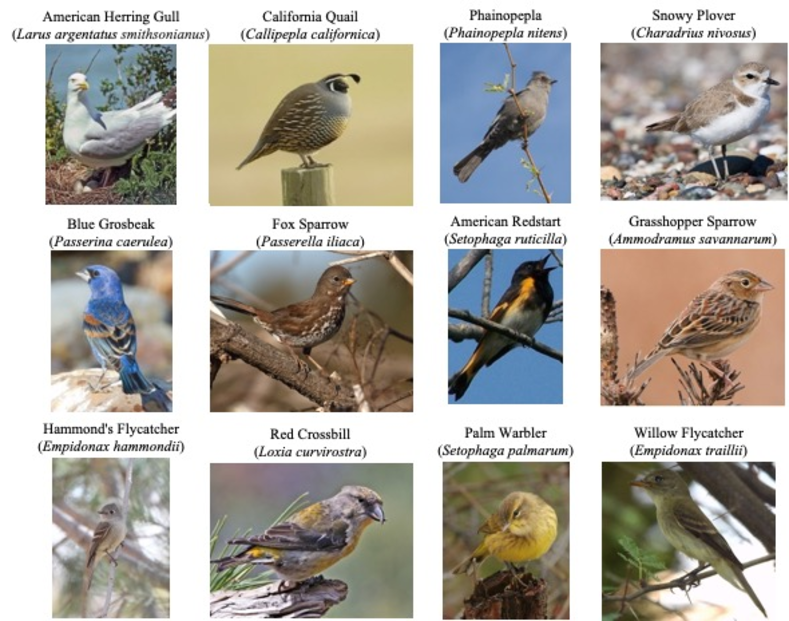

Supplement: S4 Fig — All photographs used are under the Creative Commons (CC) License. (TIF) [file pone.0289949.s004.tif]
