# Supplementary material for: Genetic identification of avian samples recovered from solar energy installations
Source: PLoS One. 2023 Sep 6;18(9):e0289949. doi: 10.1371/journal.pone.0289949 (PMC10482291; doi:10.1371/journal.pone.0289949)
Supplement: S1 Table — Numbers and sites where samples were collected. Three types of technology are represented at these sites: Photovoltaic (PV, n = 5), Concentrated Solar Power Tower (PT, n = 1), and Concentrated Solar Power Parabolic Trough (CST, n = 2). (PDF) [file pone.0289949.s005.pdf]

	Site Name	Technology	Years	Total Samples	Morphologically Unidentified Samples	Morphologically Unidentified Samples (%)
1	Arlington Solar Project	PV	2020-2021	10	1	10.0%
2	Blythe Solar Power Project	PV	2015-2017	196	26	13.3%
	Blythe Solar Power Project	PV	2018-2021	34	1	2.9%
3	Desert Sunlight Solar Farm	PV	2014-2018	272	65	23.9%
	Desert Sunlight Solar Farm	PV	2018-2021	15	2	13.3%
4	Genesis Solar Energy Project	CST	2013-2018	602	127	21.1%
	Genesis Solar Energy Project	CST	2018-2020	87	7	8.0%
5	Imperial Solar Energy Center West	PV	2016-2019	32	3	9.4%
6	Ivanpah Solar Electric Generating System	PT	2014-2018	1,320	97	7.3%
	Ivanpah Solar Electric Generating System	PT	2018-2020	1,355	432	31.9%
7	McCoy Solar Energy Project	PV	2009-2018	234	26	11.1%
	McCoy Solar Energy Project	PV	2019-2021	8	0	0.0%
8	Mojave Solar Project (Abengoa)	CST	2013-2017	218	7	3.2%
	**TOTAL**			**4,383**	**794**	**18.1%**
