# Supplementary material for: Genetic identification of avian samples recovered from solar energy installations
Source: PLoS One. 2023 Sep 6;18(9):e0289949. doi: 10.1371/journal.pone.0289949 (PMC10482291; doi:10.1371/journal.pone.0289949)
Supplement: S2 Table — Samples were categorized into Terrestrial Birds, Waterbirds, and Songbirds for this study. (PDF) [file pone.0289949.s006.pdf]

**Terrestrial Birds**	**Songbirds**	**Waterbirds**
Accipitriformes	Passeriformes	Anseriformes
Apodiformes		Charadriiformes
Caprimulgiformes		Gaviiformes
Columbiformes		Gruiformes
Coraciiformes		Pelecaniformes
Cuculiformes		Podicipediformes
Falconiformes		Suliformes
Galliformes		
Piciformes		
Strigiformes		
