# Supplementary material for: Genetic identification of avian samples recovered from solar energy installations
Source: PLoS One. 2023 Sep 6;18(9):e0289949. doi: 10.1371/journal.pone.0289949 (PMC10482291; doi:10.1371/journal.pone.0289949)
Supplement: S3 Table — Avian specimens used in the blind test. Specimens that did not match between morphological and genetic methods are highlighted by an * (see Table 2). (PDF) [file pone.0289949.s007.pdf]

	Species		Species
Specimen 1	American White Pelican (*Pelecanus erythrorhynchos*)	Specimen 25	Sora (*Porzana carolina*)
Specimen 2	Brown-headed Cowbird (*Molothrus ater*)	Specimen 26	Great Egret (*Ardea alba*)
Specimen 3	Blue-winged Teal (*Spatula discors*)	Specimen 27 *	Blue-gray Gnatcatcher (*Polioptila caerulea*) / Black-tailed Gnatcatcher (*Polioptila melanura*)
Specimen 4	Northern Shoveler (*Spatula clypeata*)	Specimen 28	Great Horned Owl (*Bubo virginianus*)
Specimen 5	Yellow-headed Blackbird (*Xanthocephalus xanthocephalus*)	Specimen 29	Red-winged Blackbird (*Agelaius phoeniceus*)
Specimen 6	Brown-headed Cowbird (*Molothrus ater*)	Specimen 30	Green-tailed Towhee (*Pipilo chlorurus*)
Specimen 7	Ash-throated Flycatcher (*Myiarchus cinerascens*)	Specimen 31	Ring-billed Gull (*Larus delawarensis*)
Specimen 8	Cinnamon Teal (*Spatula cyanoptera*)	Specimen 32	Black-throated Sparrow (*Amphispiza bilineata*)
Specimen 9	Long-eared Owl (*Asio otus*)	Specimen 33	Northern Shoveler (*Spatula clypeata*)
Specimen 10	Black Phoebe (*Sayornis nigricans*)	Specimen 34	Northern Mockingbird (*Mimus ployglottos*)
Specimen 11	Clark's Grebe (*Aechmophorus clarkii*)	Specimen 35 *	Hooded Merganser (*Lophodytes cucullatus*) / Red-breasted Merganser (*Mergus serrator*)
Specimen 12 *	Great-tailed Grackle (*Quiscalus mexicanus*) / Brewer's Blackbird (*Euphagus cyanocephalus*)	Specimen 36	Common Loon (*Gavia immer*)
Specimen 13	Red-breasted Merganser (*Mergus serrator*)	Specimen 37	Greater Roadrunner (*Geococcyx californianus*)
Specimen 14	Turkey Vulture (*Cathartes aura*)	Specimen 38	Barn Swallow (*Hirundo rustica*)
Specimen 15	Great Blue Heron (*Ardea herodias*)	Specimen 39	American Pipit (*Anthus rubescens*)
Specimen 16 *	House Finch (*Haemorhous mexicanus*) / Brown-headed Cowbird (*Molothrus ater*)	Specimen 40	Purple Martin (*Progne subis*)
Specimen 17	Lesser Nighthawk (*Chordeiles acutipennis*)	Specimen 41	American Coot (*Fulica americana*)
Specimen 18 *	Mountain Bluebird (*Sialia currucoides*) / Western Bluebird (*Sialia mexicana*) or Mountain Bluebird (*Sialia currucoides*)	Specimen 42	Black-crowned Night-Heron (*Nycticorax nycticorax*)
Specimen 19	Double-crested Cormorant (*Nannopterum auritum*)	Specimen 43	Lesser Goldfinch (*Spinus psaltria*)
Specimen 20 *	Bell's sparrow (*Artemisiospiza belli*) / Black-throated Sparrow (*Amphispiza bilineata*)	Specimen 44	Cinnamon Teal (*Spatula cyanoptera*)
Specimen 21	Rock Wren (*Salpinctes obsoletus*)	Specimen 45	White-faced Ibis (*Plegadis chihi*)
Specimen 22	Brewer's Blackbird (*Euphagus cyanocephalus*)	Specimen 46	Vaux's Swift (*Chaetura vauxi*)
Specimen 23	Western Meadowlark (*Sturnella neglecta*)	Specimen 47	Cooper's Hawk (*Accipiter cooperii*)
Specimen 24	American Avocet (*Recurvirostra americana*)	Specimen 48	Townsend's Solitaire (*Myadestes townsendi*)
